# The impact of role modelling on the future general practitioner workforce: a systematic review

**DOI:** 10.1080/14739879.2022.2079097

**Published:** 2022-07-29

**Authors:** Elizabeth Lamb, Bryan Burford, Hugh Alberti

**Affiliations:** School of Medical Education, The Medical School, Newcastle University, Newcastle upon Tyne, UK

**Keywords:** Medical education, primary care, general practice, role modelling, future GP workforce

## Abstract

Role modelling has been identified as an important phenomenon in medical education. Key reports have highlighted the ability of role modelling to support medical students towards careers in family medicine although the literature of specific relevance to role modelling in speciality has not been systematically explored. This systematic review aimed to fill this evidence gap by assimilating the worldwide literature on the impact of role modelling on the future general practitioner (GP) workforce. A systematic search was conducted in Medline, Embase, Scopus, Web of Science, Cochrane, ERIC and CINAHL, and all authors were involved in the article screening process. A review protocol determined those articles selected for inclusion, which were then quality assessed, coded and thematically analysed. Forty-six articles were included which generated four broad themes: the identity of role models in general practice, role modelling and becoming a doctor, the impact of role modelling on attitudes towards the speciality, and the subsequent influence on behaviours/career choice. Our systematic review confirmed that role modelling in both primary and secondary care has a crucial impact on the future GP workforce, with the potential to shape perceptions, to attract and deter individuals from the career, and to support their development as professionals. Role modelling must be consciously employed and supported as an educational strategy to facilitate the training of future GPs.

## Introduction

As the world recovers from the Covid-19 pandemic, primary care systems globally will shoulder an increasing healthcare burden. Doctors with a generalist skillset and aspirations to work in general practice or family medicine will be in significant demand, and it is crucial to consider the factors that may influence and deter from these ambitions.

Many variables influence individuals’ decisions to choose a career in family medicine, including workload, lifestyle preferences and organisational influences [1], in addition to valuing long-term doctor–patient relationships and the ability to see a broad spectrum of patients [2]. The influence of role models on the choice of medical career has long been recognised [3,4]. The United Kingdom (UK) reports: ‘Destination GP’ [5] and ‘By choice not by chance’ [6] highlighted the importance of role models in supporting individuals towards careers in family medicine. However, literature of specific relevance to role modelling in the speciality has not been systematically explored.

A 2013 systematic review of role modelling in medical education [4] confirmed the impact of this educational phenomenon on career choice, discussing attributes of positive role models, positive and negative role modelling, and the influence of culture, diversity and gender on the choice of role model. Whilst these findings have relevance across all medical specialities, little is known about role modelling specifically in the context of primary care medical education. The aim of this systematic review was to explore the worldwide literature focussing on the impact of role modelling in preparing the future family medicine workforce.

For the purposes of this review, the following definition of a role model has been selected: ‘a significant person on which an individual patterns his or her behaviour in a particular social role, including adopting appropriate similar attitudes’ [7]. Role models function as ‘active cognitive constructions devised by individuals to construct their ideal or possible selves, based on their own developing needs and goals’ [8]. Therefore, a medical role model is any doctor with the potential to influence the attitudes, career choice and professional development of the individuals exposed to their example.

The role modelling process is complex: individuals learn from role models through an active process of engagement, appraisal and selection of those suggestions, which are relevant to them, and construction of knowledge from the experience [9]. Social learning theory describes how people learn from each other through observation, imitation and modelling [10], doctors learn their profession through imitation of clinicians they respect and trust. They do this through conscious and unconscious observation and reflection, incorporating observed actions into their own values and behaviours [11]. Role modelling in medical education takes place in the formal, informal and hidden curricula, where learning is ‘captured, fixed and made manifest in how doctors actually set about the tasks of medicine’ [12]. Studies have discussed the attributes of positive medical role models, which can be broadly grouped into clinical competence, teaching skills and personal qualities, such as compassion, honesty and integrity [3,13–16]. Given the potential of this educational phenomenon to influence the future medical workforce, in a climate of increasing demand for generalist clinicians, it is crucial that positive role modelling in family medicine is facilitated and areas for intervention identified.

## Review objectives

This review aimed to contribute to the literature described above by answering the following questions:

1. What is the impact of role modelling in medical education in preparing the future family medicine workforce?
2. How do role models influence attitudes towards careers in family medicine or general practice?
3. What attributes are described in general practitioner role models and what impact do they have on professional skills development?

### Review methodology

Systematic review was selected as an appropriate method to assimilate the literature, aiming to systematically search for, appraise and synthesise research evidence [17], to draw together all available knowledge on the research topic. The review methodology was based on recommendations by the Best Evidence Medical Collaboration [18]. The review protocol (Appendix 1) demonstrates the inclusion and exclusion criteria, which were developed using the Population, Intervention, Comparator and Outcome (PICO) framework outlined in Table 1.

**Table 1.** PICO framework used to develop review criteria.

Population	Undergraduate medical students, junior doctors, speciality trainees
Intervention	Role models/role modelling
Comparator	Not applicable
Outcome	Impact on future workforce, career choice, attitudes towards general practice

Box 1 displays the search terms, databases used, inclusion criteria and process. This strategy was developed with the assistance of the Faculty of Medical Sciences librarian.

**Box 1.** Review search terms, databases, inclusion criteria and process

Review search terms: General practice; general practitioner; primary healthcare; family doctor; family practice; primary care AND role model; role modelling AND medical education; medical students; medical schools; junior doctors; registrar; GP trainee; graduate medical education; internship; residency
Databases searched: Medline, Embase, Scopus, Web of Science, Cochrane, ERIC and CINAHL. Hand searches of Medical Teacher, Education for Primary Care, British Journal General Practice and British Medical Journal were also undertaken. All databases were searched from 1990 to 2020 (studies published before 1990 were excluded as an initial scoping review suggested very little literature on this subject specific to GP pre-1990) and limited to English language due to lack of translation budget
Inclusion criteria (see appendix 1): All primary research studies which discussed role models and role modelling in general practice and the influence of these on either medical students or junior doctors were included. Published between 1990 and 2020.
Process: Articles identified in the search databases were uploaded to Rayyan software (https://rayyan.qcri.org/), these articles were screened based on their title and abstract. All authors were involved in article screening; where there was discrepancy in opinion EL retrieved the full text of the article and made a final decision on whether to include the article. References used by the included papers were also screened and full text retrieved in cases where the reference appeared relevant. All references were uploaded to an EndNote X9.2 library.

Included studies were coded on individual data abstraction sheets, supporting identification of the main findings of relevance to the concepts, which informed the review questions. The extracted data were then thematically analysed, following the framework described by Braun and Clarke [19]. The methodological quality of each included study was then assessed using a series of quality indicators validated in the Best Evidence in Medical Education guide 11 [18] and used in a systematic review discussing doctor role modelling in medical education [4], see Appendix 2.

## Results

**Figure 1.**
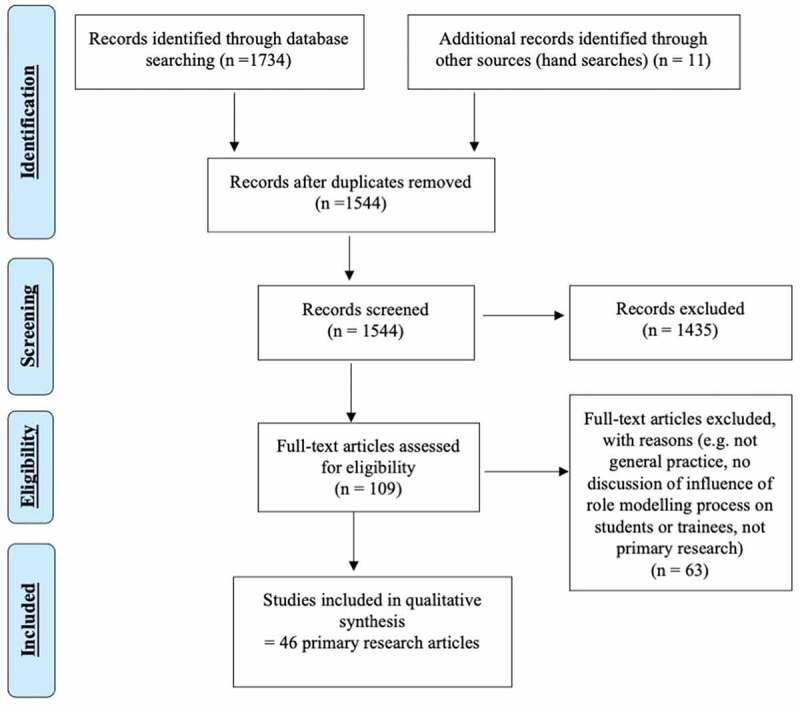
Prisma 2009 flow diagram [20] detailing the article selection process.

Figure 1 demonstrates the article selection process, which adhered to the PRISMA checklist [20]. In total, 1,544 articles were screened, leading to a selection of 46 primary research papers. Each article was summarised on an Excel spreadshee. Higher quality studies were considered to have met at least nine out of 11 of the quality indicators, medium quality seven or eight, and lower quality papers those that scored six or below.

Most of the included articles were from the UK and North America, with others from Australia, Germany, the Netherlands, Japan, Ghana, Finland, Switzerland, and Saudi Arabia. Most papers were published between 2000 and 2020, with five published in the 1990s. Thirty-one primary research papers were rated as high quality, 14 were medium and one low quality. The most common research method was survey, followed by interviews and focus groups. Thematic analysis of included articles and discussion between all authors led to the development of four broad themes, which were as follows: the identity of role models in general practice, role modelling and becoming a doctor, the impact of role modelling on attitudes towards the speciality and the subsequent impact on behaviours/career choice. Table 2 summarises the included articles and themes.

**Table 2.** A summary of the included articles and their allocated themes.

Author/Title	Year	Country	Method	Conclusions	Theme	Score
Biringer, A; Forte, M et al What influences success in family medicine maternity care education programs?	2018	Canada	Semi structured interviews with 18 participants	Credible role models support belief that low risk obstetric care is within scope of family medicine	2	10
Burack J, H; Irby, D A study of medical students speciality choice pathways, trying on possible selves	1997	USA	Survey of 157 students, focus groups with 47 students	The process of speciality choice can be described as a socially constructed process of ‘trying on possible selves’	1	9
Delva, D; Kerr, j Continuity of care, differing conceptions and values	2011	Canada	Semi-structured interviews with 30 participants	Different perceptions, settings, and skills can influence how continuity of care is valued, trainees learn about continuity through role models.	1, 2	7
Deutsch, T; Honigschmid, P Early community-based family practice elective positively influences medical students’ career considerations–a pre-post-comparison	2013	Germany	Survey of 140 students	Exposure to primary care role models early in training encourages students to consider family practice as a career option.	3	9
DeWitt, D.E; Curtis, J. What influences career choices among graduates of a primary care training program	1998	USA	Survey and semi-structured interviews with 88 participants	A substantial minority of primary care residents pursue speciality careers rather than primary care, lack of positive primary care role models cited as one of the reasons for this career change.	4	8
Essers, G.Van Weel-Baumgarten, E.Bolhuis, S. Mixed messages in learning communication skills? Students comparing role model behaviour in clerkships with formal training	2012	The Netherlands	Survey of 289 students	Role models in general practice are not reliably demonstrating the sort of communication taught during undergraduate training.	1	8
Jordan, J; Brown, J et al. Choosing family medicine. What influences medical students?	2003	Canada	Semi-structured interview with 11 participants	Exposure to GP role models in training validate and reinforce students’ decision to go into FM. Role models demonstrate importance of patient-doctor relationship and continuity of care.	2,4	9
Knox, K; Getzin, A et al. Short report: factors that affect speciality choice and career plans of Wisconsin’s medical students	2008	USA	Survey of 304 students	Students choosing primary care are more likely to be influenced by role models than those pursuing other specialities, particularly for males	4	8
Mann, K et al Community family medicine teachers’ perceptions of their teaching role	2001	Canada	Interviews of 17 teachers	Teachers’ perceptions of their role, importance of conscious role modelling, demonstrating skills, attitudes, knowledge	1	7
Matthews, C Role modelling: how does it influence teaching in Family Medicine?	2000	Saudi Arabia	Semi-structured interviews and survey with 15 teacher participants	The four best-remembered teacher behaviours were: positive behaviour towards patients, negative behaviour towards junior colleagues, effective presentation of subject content and encouragement to participate in patient care.	1, 3	4
Meli, D. N.et al General practitioner teachers’ job satisfaction and their medical students’ wish to join the field – a correlational study	2014	Switzerland	Survey of 184 student/ teacher participants	Medical students’ perception of their GP teachers’ job satisfaction positively affect their wish to become GPs, and their satisfaction with their internships adds to this.	3,4	9
Schafer, S et al Rejecting family practice: why medical students switch to other specialities	2000	USA	Survey of 320 medical graduates	Students rejecting family practice were more likely than their colleagues rejecting other specialities to cite insufficient prestige, low intellectual content, and concern about mastering too broad a content area as reasons.	3	8
Nicholson, S et al. Influences on students’ career decisions concerning general practice: a focus group study	2016	UK	Focus groups of 58 students	Early, high-quality, ongoing and, authentic clinical exposure promotes general practice and combats negative stereotyping.	3,4	11
Reitz et al. Balancing the Roles of a Family Medicine Residency Faculty: A Grounded Theory Study	2016	USA	Interviews with 121 family medicine teachers	The perspective of GP educators, confirm that being a role model is a primary role of family medicine educators	1	11
Risenberg, L,A et al. Medical student and faculty perceptions of desirable primary care teaching site characteristics	2001	USA	Q sort exercise/ survey of 39 students and 20 educators	Preceptors make the difference in primary care educational experience, though teachers tend to see role modelling as more important than the students.	1	7
Saigal, P et al. Factors considered by medical students when formulating their speciality preferences in Japan: findings from a qualitative study	2007	Japan	Interviews with 25 students	Role models are reported by Japanese students as influential factors when formulating their speciality preferences.	4	7
Silverstone et al. Students’ conceptual model of a good community attachment	2001	UK	Focus groups with 31 students	A theoretical model based on student conceptualisation of ‘a good GP’ was developed consisting of the GP as a teacher, as a role model and as an indicator of a positive learning environment.	1	9
Vohra, A.et al. Factors that affect general practice as a choice of medical speciality: implications for policy development	2019	Australia	Interviews with 47 participants ranging from students to practicing GPs	Effect of role models found to be a key factor affecting choice of speciality- lived experiences and personal relationships with individuals had a lasting effect on participants choices of speciality.	4	10
Wiener-Ogilvie, S et al. Foundation doctors career choice and factors influencing career choice	2015	UK	Survey of 543 foundation doctors	Undergraduate GP placement was reported as the strongest influence in favour of a career in General Practice	4	8
Henderson, E et al. Attitude of medical students towards general practice and general practitioners	2002	UK	Survey of 700 students	Personal experience of GP role models is the most important factor influencing attitudes to GP which improve over the course of their training.	3	10
Ambrozy, D. M et al. Role models’ perceptions of themselves and their influence on students’ speciality choices	1997	USA	Survey of 177 student identified faculty role models	The role models in this study agreed with their students about what is important to model including demonstrating enthusiasm and a sincere love for what they do.	1	8
Barber, et al. UK medical students’ attitudes towards their future careers and general practice: a cross-sectional survey and qualitative analysis of an Oxford cohort	2018	UK	Survey of 280 students	Medical students may be put off careers in general practice by three main things: low perceived value of community-based working and low status of general practice, observing the pressures under which GPs currently work; and lack of exposure to academic role models and primary care-based research opportunities.	1,3	10
Bien, A et al. What influence do courses at medical school and personal experience have on interest in practicing family medicine? – Results of a student survey in Hessia	2019	Germany	Survey of 361 students	Positive role models influence attitudes towards GP and subsequent career choice.	3,4	9
Campos-Outcalt, D.; Senf, J.et al A Comparison of Primary Care Graduates from Schools with Increasing Production of Family Physicians to Those from Schools with Decreasing Production	2004	USA	Survey of 1457 graduates	Positive and competent family medicine role models encourage students towards careers in family medicine	4	7
Connelly, M, T; Sullivan, A.M et al Variation in predictors of primary care career choice by year and stage of training: A national survey	2003	USA	Survey of 1665 students and residents	Primary care role models are a significant predictor for entering a career in primary care	4	10
Deutsch, T.; Honigschmid, P.et al Early community-based family practice elective positively influences medical students’ career considerations–a pre-post-comparison	2013	Germany	Survey of 140 students	Exposure to primary care role models early on in training encourages students to consider family practice as a career option.	4	9
Elnicki, D. M.; Kolarik, R et al. Third-year medical students’ perceptions of effective teaching behaviours in a multidisciplinary ambulatory clerkship	2003	USA	Survey of 276 students	Teaching effectiveness in primary care was found to be independently associated with providing a role model.	1	7
Firth, A.; Wass, V.; Medical students’ perceptions of primary care: The influence of tutors, peers and the curriculum	2007	UK	Open in depth interviews with 11 students	Poor-quality attachments and negative role modelling of primary care reinforced negative views and impacted detrimentally on career choice	1, 3	11
Jochemsen-Van Der Leeuw, H. G. A. R.; Van Dijk, N.et al Assessment of the clinical trainer as a role model: A role model apperception tool (RoMAT)	2014	Netherlands	Survey of 279 GP trainees	The RoMAT proved to be a valid, reliable instrument for assessing clinical trainers’ role-modelling behaviour.	1	11
Kutob, R. M.; Senf, J. H.;The diverse functions of role models across primary care specialities	2006	USA	Survey of 1457 physicians	For family medicine and internal medicine graduates, having a role model was related to more contact and more-positive views of faculty in their speciality. Those with a role model reported that primary care was encouraged at their medical school and were more satisfied with their speciality choice.	1, 4	10
Miettola, J.; Mantyselka, P.; et al Doctor-patient interaction in Finnish primary health care as perceived by first year medical students	2005	Finland	Written reports from 127 students	Medical students’ perceptions in this study support the importance of role models (good or bad) in making good doctors. Students saw witnessing poor role modelling as an opportunity for learning.	1, 2	9
Moyer, C. A.; Arnold, L.;et al What factors create a humanistic doctor? A nationwide survey of fourth-year medical students	2010	USA	Survey and focus groups with 80 students	Students viewed a variety of factors as influencing their development of humanism, with role models being of greatest importance	2	10
Mutha, S.; Takayama, J et al Insights into medical students’ career choices based on third- and fourth-year students’ focus-group discussions	1997	USA	Focus groups with 52 students	Role models affect speciality choice, negative role models, based on the students’ assessments of interpersonal interactions and career satisfaction, were particularly influential in closing doors to certain fields.	3, 4	11
Nicol, J. W. Gordon L.J. Preparing for leadership in General Practice: a qualitative exploration of how GP trainees learn about leadership	2018	UK	Interviews with 15 GP trainees	Role modelling important part of informal curriculum in learning about leadership. Fallibility of leaders can positively influence learning	2	11
Reid, K.; Alberti, H.; Medical students’ perceptions of general practice as a career; a phenomenological study using socialisation theory	2018	UK	Focus groups with 14 students	Medical students perceive general practice to lack prestige and challenge. These perceptions come, at least in part, from a process of socialisation within medical school, whereby medical students internalise and adopt their role models’ perceptions and values, and the values portrayed by the hidden curriculum in their medical school culture.	1,2,3	10
Rodríguez, Charo, López-Roig, Sofía et al. The Influence of Academic Discourses on Medical Students’ Identification With the Discipline of Family Medicine	2015	Canada/ France/ Spain/ UK	Focus groups with 132 students and semi-structured interviews with 67 educators	In UK perceptions of GP positive and role models mentioned in the formation of positive perceptions, in other countries disgruntled role models had a negative impact on perceptions.	3	11
Alberti, H. Banner, K.et al Just a GP’: A mixed method study of undermining of general practice as a career choice in the UK	2017	UK	Survey of 780 foundation doctors and focus groups with 49 foundation doctors	GP role models consistently a positive factor influencing participants and other clinicians’ perceptions of GP. Role models in hospital specialities making negative comments about GP.	3,4	11
Ie, K. Tahara, M. Factors associated to the career choice of family medicine among Japanese physicians: The dawn of a new era	2014	Japan	Survey of 41 primary care physicians	Family medicine role models in Japan are important in choosing a career in family medicine, but lack of role models is an issue	4	7
Lublin, J. R Role modelling, a case study in general practice	1992	Australia	Survey and interviews, 22 GPs and 93 students	Modelling good patient relationships was most the most desirable attribute for students, and students admired skill and knowledge, role modelling is a powerful influence on students.	1	9
Amalba, A. Abantanga, F. Community-based education: The influence of role modelling on career choice and practice location	2017	Ghana	Survey of 149 students	The desire and willingness to work in a rural community combined with good communication and excellent interpersonal skills as well as good leadership skills are attributes of good role models with potential to influence medical students’ career choice.	1, 2	10
Dallas, A. Van Driel, M. Antibiotic prescribing for the future: exploring the attitudes of trainees in general practice	2014	Australia	Semi-structured interviews and focus groups with 17 GP trainees	Role models in primary care important in shaping antibiotic prescribing habits.	2	10
Passi V, Johnson, N The impact of positive doctor role modelling	2016	UK	Focus groups with 52 medical students and semi-structured interviews with 5 students and 30 consultants	Role models are critically important in the professional development, character development, and career development of modelees. Role modelling effectively enhances the transformation of the student to a doctor.	2, 4	11
Roos, M, Watson, J et al Motivation for career choice and job satisfaction of GP trainees and newly qualified GPs across Europe: a seven countries cross-sectional survey	2014	Germany	Survey of 3722 GP trainees and newly qualified GPs	Role models influence career choice in male rather than female participants.	4	10
Curtis, A. Main, J. ‘Getting them early’: the impact of early exposure to primary care on career choices of A-level students – a qualitative study	2008	UK	Interviews with 11 students	Role models are important in the professional development of medical students	3	8
Passi V, Johnson, N The hidden process of doctor role modelling	2016	UK	Focus groups with 52 medical students and semi-structured interviews with 5 students and 30 consultants	This research study generated a detailed explanation of the process of doctor role modelling and how this subsequently influences professional development.	2	11
Mackie, E. Alberti, H. Longitudinal GP placements- inspiring tomorrow’s doctors	2020	UK	Semi-structured interviews with 5 students	The GP tutor role model and ‘authentic’ experiences to consult patients themselves developed a growing sense of self-efficacy within students, all of which resulted in a significant internal desire to become future GPs.	1, 4	10

### Theme one: the identity of role models in family medicine

In this first theme, we discuss what makes a family medicine doctor, or general practitioner (GP) a role model, in what context they act and their attributes. Role models are crucial in shaping future doctors, who are particularly likely to be influenced by role models who match their personalities, values and represent ‘possible selves’ [21]. Positive role models appear to be of particular importance in influencing those who choose to specialise in family medicine, less so for those who elect to specialise in secondary care. Role models play a vital role in helping students to understand the role of a GP, making explicit the values of the speciality and supporting students who share those values towards a career in general practice [22].

GP educators are commonly identified as role models [23], longitudinal community placements are an ideal context for exposure to the role modelling process [24], and GP trainees are described as effective role models acting as powerful advocates for the role [25]. GPs in the media, direct personal experience of a GP as a patient, and having a GP parent were identified as role modelling encounters [26].

The explored literature provides limited insight into differences in individual characteristics such as gender and ethnicity in the identification of a role model. There is conflicting information on the link between gender and the identification of GP role models, with some studies suggesting men are more likely than women to cite role models in influencing their decision to choose general practice [27,28], and others suggesting women are more likely to identify a GP role model [22]. Individuals from some minority and white ethnic groups may be less likely to identify a role model [22].

Positive role model attributes described in the literature include demonstrating enthusiasm towards patients, students and teaching; dedication to the career; creating a safe and comfortable environment; being open to reveal the ‘human’ behind the clinician and honesty about the challenges faced [24]. Key to providing a positive role model are good communication skills, non-judgemental attitudes [29], showing empathy [21], demonstrating holistic care and being knowledgeable and genuinely interested in patients [30–32]. Positive role models are visionary leaders [33] who are compassionate, inspiring [34] organised [26], demonstrate intellectual curiosity and a good work–life balance [23].

Negative behaviours were witnessed in GP role models, including taking shortcuts in examination skills, treating ‘patients like a production line’, demonstrating prejudice, failing to interact with students [26], poor interpersonal relationships [34] and poor communication skills [31]. Furthermore, GPs were witnessed ‘bashing’ their own speciality [35].

Medium and low scoring papers reinforced the findings described above around communication skills [36]. In addition, teaching effectiveness was found to be independently associated with providing a good role model [37], and conscious role modelling was deemed a professional responsibility of educators [38,39]. GP role models can have an important impact before medical school during student work experience, raising awareness of the speciality to school pupils [40]. Additional positive attributes to those detailed above include providing continuity of care [41] and demonstrating clinical reasoning skills [42].

### Theme two: role modelling and becoming a doctor

The review identified several high-quality papers discussing the impact of role modelling on professional development of future doctors and developing the skillset of a GP. Doctor role modelling in medical education is critically important in the professional development, character development and professional identity formation of future doctors, effectively ‘enhancing the transformation of the student to a doctor’ [43]. Participants in this study developed an idea of what ‘type’ of doctor they would like to become, not just what speciality they will choose, through role modelling and emphasised the value of ‘emulating the role model’s unique approach and styles’. A subsequent grounded theory study led by the same authors [44] generated an explanation of the process of modelling and how it leads to behaviour change through exposure to the role model, critical appraisal of the behaviours observed and then a model-trialling cycle of behaviours.

Students undergo a socialisation process at medical school where they transition from a lay person to a doctor. Crucial in this process is the role model, whose values they internalise and adopt, shaping their professional identity [35]. Role models are of utmost importance in the development of humanism [45], and through role modelling, future doctors develop professional skills including doctor–patient relationship, continuity of care and prescribing habits [41,46,47]. Role models demonstrate the opportunity for portfolio careers and special interests, such as obstetric care [48]. Role modelling is important in developing leadership skills [33]. Interestingly, witnessing poor leadership and navigating the strains of professional life can provide as rich an opportunity for learning as exposure to good leadership role models. Indeed, this learning opportunity from negative experiences is key in creating ‘good doctors’ [31].

### Theme three: the impact of role models on attitudes towards family medicine

Role models play a critical role in forming perceptions of family medicine. Personal experience of GPs has been identified as the most important factor influencing attitudes of undergraduates to the speciality [49], while secondary care clinicians can play a significant role in development of negative perceptions of the speciality [35]. GP role models often promote the speciality, combat negative stereotyping and increase understanding of what it means to be a ‘good GP’ [22,50]. Witnessing the job satisfaction of role models in providing continuity of care and caring for patients with complex co-morbidity shapes a positive view of the profession [51,52].

A large international study [53] highlighted the importance of GP role models in contributing to undergraduate perceptions, describing that through witnessing positive GP role models, UK students perceived a career in family medicine to provide autonomy, diversity of practice, a good quality of life and work–life balance. However, in Canada, France and Spain, family medicine was not as well regarded, and disgruntled role models had a negative impact on perceptions. This positive UK experience is contradicted to some degree by a survey-based study, which explored perceptions of UK medical students around careers in general practice. Many respondents cited particular GPs shaping perceptions that GPs have lower status than hospital specialists, that the role lacks intellectual challenge, limited research opportunities, and that GPs were stressed, lacked autonomy and unfulfilled [25]. Further studies [35,54] confirm that role models contributed to the perception that family medicine lacked prestige and challenge, and some role models were perceived to be dissatisfied with their career.

Attitudes were affected through role models outside of training in general practice. Participants with GP parents in a UK-based study described the negative impact of witnessing them working long hours, and GP role models in the media were seen as ‘not actually doing anything’ and not mentioned in ‘exciting’ dramas [26]. Role models in the media seem to be particularly influential to early-stage medical students, perhaps because they have less opportunity for engagement with real GP role models, who are responsible for improving perceptions of the speciality as they progress through training [49]. Role models in factual media contributed to negative perceptions, and Harold Shipman was mentioned by many students as influencing their attitude towards the speciality [26].

Concerningly, hospital role models are influential in shaping negative perceptions of family medicine [26] and GP trainees witnessed secondary care role models commenting on the speciality being boring, ‘a waste of training’ and a ‘second-class career choice’, reinforcing a negative perception of being ‘just a GP’ [55]. Students perceived the job of a GP as ‘difficult to do well’ as they witnessed secondary care role models ‘bashing GPs’ and their ‘poor’ or ‘inappropriate referrals’ [35]. Medium and low scoring papers provide further evidence of role modelling influencing the perceptions of future doctors [40,56] and role models contributing to a perceived lack of intellectual challenge in the speciality [57].

## Theme four: the influence of role modelling on career choice

Role modelling is crucial in shaping the attitudes of the future workforce towards general practice. This section summarises findings from the included literature discussing the influence of role modelling on choosing a career in general practice.

Having a GP role model is a significant predictor of entering a career in the speciality, an effect that at the time of this study appeared to be stronger in junior doctors than students [58]. Those who have chosen the speciality and identify a GP role model are more likely to have trained at a university where family medicine was encouraged, and individuals were more likely to be satisfied with their career choice [22]. Exposure to GP role models through high quality, early placements positively influences students’ views on a career in the speciality [50,51,59,60] and relationships with GP role models have a lasting effect on a decision to choose a career in general practice [46,55,61]. GP role models are crucial in a matching process between understanding the role of a GP and increasing understanding of the self, resulting in a desire to enter family medicine [24].

Witnessing the administrative burden in GP role models, negative behaviours [26,54], appearing dissatisfied with their job [52] and secondary care role models [55] all have the potential to deter future doctors from a GP career. Medium and low scoring papers confirmed that role models attract [62,63] and deter trainees from pursuing a career in the speciality [64,65]. Role models appear to be of greater importance in influencing career choice towards primary care than secondary care specialities, particularly for male students [66].

## Discussion

**Figure 2.**
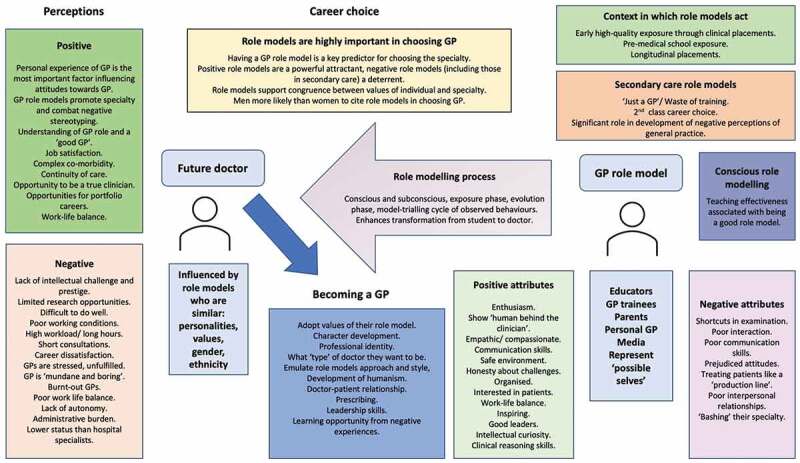
A summary of the attributes of GP role models and the influence of the role modelling process on perceptions, behaviours and career choice.

Our systematic review has confirmed the importance of role modelling in influencing the future family medicine workforce, highlighting that the role modelling process not only affects attitudes to and career decisions towards the speciality but also affects the process of becoming a doctor. We have included the findings from 46 relevant primary research articles from around the world and of particular importance, we have identified the powerful impact of denigration of general practice from role models in both primary and secondary care, and the perceived lack of intellectual challenge and research opportunities in the speciality. We have confirmed the importance of students being exposed to GP role models, ideally through high quality, longitudinal placements. We have described the importance of the GP role model in developing professional identity in future doctors, shaping their understanding of the values of the speciality, development of humanism, leadership and communication skills. Figure 2 summarises the review findings.

Our findings on the influence of GP role models on the professional development of future doctors are supported by a systematic review of undergraduate medical education in general practice [67], which identified a vital component of training as observing role models in action, allowing students to observe, think and develop professional attitudes and behaviours, which mirror their role models. According to social learning theory [10] individuals pay attention to role models because they believe they can learn professional skills and attributes from them, particularly if they observe behaviour aligned with their views of what is important about being a doctor. Therefore, it is crucial that GPs involved in teaching role model consciously, and demonstrate appropriate attitudes and behaviours, as they have the potential to have a profound influence on the attributes of the future medical workforce.

Our review identified that some medical students and trainees were deterred from a career in general practice as they perceived GPs to be stressed with high workloads and a job that was difficult to do well. An international review highlighted that GPs were particularly prone to burnout and that burnout appeared to be frequent amongst those training in the speciality [68]. We found that GP trainees can be particularly effective and positive role models. Reports suggested medical schools worldwide now have formal arrangements for GP trainees to teach medical undergraduates [69–72]. Trainees are often more cognitively and socially congruent with their learners [73] and using near-peer role models to teach medical students is encouraged in the recommendations made in ‘By Choice not by Chance’ [6]. However, trainees face stress and workload pressures, and may be more susceptible to burnout. A study that analysed the personality traits of GP trainers compared with GP trainees found that trainees reported lower levels of emotional resilience [74], and a Hungarian study identified moderate-to-high levels of emotional exhaustion and depersonalisation in both groups but higher in trainees [75]. While those considering a career in the speciality need to see the realities of the role, it is important to consider how to support the GP workforce to remain positive advocates for their career while also role modelling a positive approach to the realities of the job and the stressors entailed.

We identified that role models in general practice may be perceived to have lower status than hospital specialists, with limited academic opportunities, and students witnessed GP role models ‘bashing’ their own speciality. The influence of secondary care clinicians in promoting the idea that general practice is a second-rate career is concerning. The motivational theory of role modelling [76] described that role models represent the possible and provide inspiration. Lack of academic possibilities and negativity towards the speciality is likely to de-motivate students from aspiring to a career in general practice. Future doctors are high achieving, highly intelligent individuals, and if they perceive general practice to be boring and not academic, it is questionable whether they will identify with that role.

Evidence has suggested that individuals were more likely to identify role models who they perceive as sharing similarities to them. The social comparison theory [8] supports this finding, suggesting that individuals are drawn to people who they perceive as similar to themselves, and represent an aspect of what the individual would like to become. The interplay between intersectional identity and role models is likely to be complex. The literature describing this relationship was limited to quantitative data suggesting a correlation between gender and ethnicity and the identification of a role model. It is important that this area is further explored with qualitative work. Box 2 has suggestions for further research to explore pertinent questions around personal attributes and identity of role models amongst other key topics.

Based on our review findings, we recommend that the following steps described in Table 3 are taken to ensure role modelling is used to its full potential in supporting doctors towards careers in general practice.

**Table 3.** Implications for practice.

Conscious role modelling	Institutions must ensure that family medicine educators are aware of their role modelling potential and are supported to demonstrate appropriate professional behaviours and attributes.
Supporting positive role modelling	Consideration of the emotional status and support needs of those involved in providing a role model for future doctors is important, particularly in those who are junior and potentially at greater risk of burnout.
Training the future generalists	GP role models are ideally placed to support the development of key generalist skills therefore, all future doctors should receive regular and ideally longitudinal exposure to GPs throughout their medical training.
Exposure to GP role models	Medical students must receive appropriate exposure to GPs throughout training. Institutions should seek to support academic GP role models to raise the profile of general practice as an academic speciality and dispel the view that it lacks intellectual challenge.
Denigration of general practice	Action must be taken to highlight the detrimental impact of denigration of GP as a speciality, and professional ‘banter’ of GP bashing has no place in modern medicine.

### Strengths and limitations

This systematic review is the first we are aware of to highlight the importance of role modelling as an educational strategy in securing the future family medicine workforce. Our systematic search strategy allowed exploration of several key areas of interest enhanced by thematic analysis. This led to the development of several practical recommendations, which have the potential to impact on future GPs. However, this review had limitations. Limiting our findings to English language articles meant we may have missed other international evidence. Using role model/role modelling in our search terms meant we may have missed articles that described a role model in another way, for example, a mentor.

## Conclusion

Our systematic review highlighted the importance of the future workforce being exposed to positive GP role models to favourably shape perceptions, career choices towards general practice and the development of generalist attributes. The review highlighted the potential detrimental impact of negative role modelling by doctors in both primary and secondary care, which can deter future doctors from careers in general practice. Further steps must be taken to increase awareness of the professional responsibility as a GP and secondary care doctor to act as a positive role model to future doctors, and institutional support must be provided to role models to enable them to act consciously and positively to support the future GP workforce.

**Box 2.** Recommendations for future research

Understanding the interplay between intersectional identity, personal attributes (gender, race/ ethnicity, disability etc) and role modelling in family medicine
Exploring the experience of the GP role model and the barriers and facilitators to effective role modelling.
The lived experience of future doctors- how do individuals experience the role modelling process in general practice?

## Appendix 1: Review protocol

- Was the study published after 1990?
- Is the full text available in English language?
- Is the study a primary research article containing qualitative or quantitative data on GP role models or role modelling in general practice (or family medicine) and their influence or impact on either medical students or junior doctors?
- Exclude editorials, letters, papers which do not present primary research.
- Exclude studies discussing role modelling solely by other health professionals

Key words to include:

- General practitioner/general practice/family medicine
- Role models/role modelling
- Medical students or junior doctor or GP trainee or resident or intern or foundation doctor or registrar

Articles should describe the impact or influence of role models or the role modelling process on the above groups; these may be perceptions, career choice, professional identify or other ways in which the decisions made by these groups are influenced.

## Appendix 2: Quality assessment tool

Reviewer:

Authors:

Year:

Country and Institution:

**Table:**

Quality indicator	Score 0/1	Summary
Research question: is the question or hypothesis clearly stated?		
Study subjects: is the subject group appropriate for the study being carried out (number, characteristics, selection and homogeneity?)		
Data collection methods: Are the methods used reliable and valid for the research question and context?		
Completeness of data: Have subjects dropped out? Is the attrition rate less than 50%? For questionanaire based studies is the response rate acceptable (60% or above?)		
Control of confounding: Have multiple factors/ variables been removed or accounted for where possible?)		
Analysis of results: Are the statistical or other methods of results analysis used appropriately?		
Conclusions: is it clear that the data justify the conclusions drawn?		
Reproducibility: Could the study be repeated by other researchers?		
Prospective: Does the study look forwards in time rather than backwards?		
Ethical issues: Were all relevant ethical issues addressed?		
Triangulation: Were results supported by data from more than one source?		

Total score =
